# A Comprehensive Review of Quality of Life (QOL) Research in Hong Kong

**DOI:** 10.1100/tsw.2007.217

**Published:** 2007-08-17

**Authors:** Daniel T. L. Shek, Britta M. Lee

**Affiliations:** ^1^ Center for Quality of Life, Hong Kong Institute of Asia-Pacific Studies, The Chinese University of Hong Kong, China; ^2^ Kiang Wu Nursing College of Macau, China; ^3^ Social Welfare Practice and Research Centre, The Chinese University of Hong Kong, China

**Keywords:** quality of life, QOL research, Hong Kong

## Abstract

Published quality of life (QOL) studies in Hong Kong indexed in the major databases were reviewed. Several observations are highlighted from this review. First, most of the published studies were empirical studies involving data collection. Second, there are more micro studies utilizing individual QOL indices than macro studies using societal indicators. Third, most studies addressed personal well-being, followed by studies on family well-being and societal well-being. Fourth, the studies were predominantly quantitative in nature. Fifth, most of the studies were based on adults and comparatively fewer studies were based on children and adolescents. Sixth, most studies were based on populations with special needs, followed by studies based on the general population, helping professionals, and caregivers. Seventh, most studies used measures of QOL rather than developed QOL measures. Finally, QOL data in Hong Kong were seldom compared with those in other places. The gaps on QOL studies in Hong Kong and future research directions are discussed.

## INTRODUCTION

In many fields, including medicine, health sciences, and social sciences, the concept of quality of life (QOL) has attracted much research focus[1,2]. Although there are different definitions of the concept, there is a general agreement among researchers[3,4] that QOL is a multidimensional concept comprising material well-being (finance, income, housing quality, transport), physical well-being (health, fitness, mobility, personal safety), social well-being (personal relationships, community involvement), emotional well-being (positive affect, mental health, fulfillment, satisfaction, faith/belief, self-esteem), and productive well-being (competence, productivity). Besides, there are researchers who argue that objective life situation (e.g., literacy rate in a society) as well as subjective perceptions of an individual in evaluating one's objective living conditions (e.g., subjective satisfaction with one's life) are important dimensions to be considered[5]. There are also views that suggest that a holistic approach is needed to understand and research the concept of "quality of life"[1,2].

In a comprehensive review of QOL studies in the Chinese contexts, Shek et al.[1,2] pointed out that there were several research gaps in the existing literature. These missing research gaps are summarized as follows:

- Gap 1: Based on an ecological understanding that posits that QOL at different systems is important, the focus on family QOL and spirituality are not adequate in the existing literature on QOL.
- Gap 2: There are relatively more QOL research studies on adulthood and old age, while there are comparatively fewer research studies on the QOL of children and adolescents.
- Gap 3: The majority of the QOL studies has been conducted in Western contexts based on Western participants and comparatively fewer QOL studies have been conducted in different Chinese contexts.
- Gap 4: There are studies addressing QOL in people in the vulnerable groups. However, while QOL studies in the medical and rehabilitation settings have commonly focused on the physically disadvantaged and intellectually impaired groups, comparatively fewer studies have examined the QOL of persons experiencing economic disadvantage.
- Gap 5: Although researchers in different disciplines, including psychology, sociology, social work, education, communication, medicine, and health-related disciplines, have examined the concept of QOL, dialogues among researchers in different professions are not extensive and forums for exchanges among different professionals are few.
- Gap 6: QOL studies involving the comparison of different countries in a single study are not widespread.
- Gap 7: With reference to the issue of how QOL at the aggregate level (e.g., community, societal, national, regional, international, or global level) can be assessed adequately, there is still the debate surrounding whether objective indicators (such as official statistics) or subjective indicators (such as opinion surveys) are better indicators of QOL.

With reference to the above-mentioned research gaps, there is a need to know the characteristics of the available QOL studies so that future research direction can be formulated. In this study, published QOL studies in Hong Kong indexed by the major databases were reviewed and the major characteristics of the published papers are highlighted.

## METHODS

Two search terms ("quality of life" and "Hong Kong") were used to search the relevant QOL studies in several databases that included: (a) PsycINFO (years 1806 – Jun/2007); (b) Sociological Abstracts (years 1963 – Jun/2007); (c) Social Sciences Citation Index (years 1956 – Jun/2007); (d) MEDLINE (years 1950 – Jun/2007); (e) CINAHL (years 1982 – Jun/2007); and (f) others including ERIC (years 1966 – Mar/2007), Educational Full Text (years Jun/1983 – Apr/2007), and Social Work Abstracts (years 1977 – Mar/2007). Papers that were not relevant to the scope of QOL research were discarded. The total counts of relevant papers reviewed in the above-mentioned databases were 121, 34, 199, 174, 95, and 29, respectively, and the final count of papers reviewed without duplication was 373.

After locating the 373 papers, they were reviewed and categorized with a classification scheme that consisted of seven major dimensions: type, scope, methodology, stages of life span, sample population, issue addressed, and comparison with other countries. Among the seven major dimensions, the papers were first classified by type: (a) papers that addressed theoretical or conceptual issues [T], and (b) papers that were based on empirical studies [E]. For papers classified as empirical studies, further classifications in the remaining dimensions were carried out.

In the second dimension, the empirical studies were first classified according to the scope of the study. There are two major categories in this dimension: (a) studies utilizing micro QOL indicators, which were further divided into (i) personal well-being domain that included aspects such as self-esteem [Per WB], (ii) family well-being domain that included aspects such as interpersonal relationships [Fam WB], and (iii) societal well-being domain that included aspects such as environment and sense of security [Soc WB]; and (b) studies utilizing macro QOL indicators.

The third dimension is related to the methodologies adopted in the empirical studies, which were classified into three categories: (a) studies that adopted quantitative approach [QT], (b) studies that adopted qualitative approach [QL], and (c) studies that adopted mixed-methods approach [MIX]. In the fourth dimension, stages of life span concerned in the empirical studies were classified into six categories: (a) studies that addressed the infant stage: ages 0–2 [INFANT], (b) studies that addressed the childhood stage: ages 3–11 [CHILD], (c) studies that addressed the adolescence stage: ages 12–17 [ADOL], (d) studies that addressed the adulthood stage: ages 18–59 [ADU], (e) studies that addressed the aged stage: ages 60 and over [AGED], and (f) studies that addressed all stages [ALL].

The fifth dimension is concerned about the sample population of the empirical studies. There were four categories of researches in this dimension: (a) studies based on samples drawn from the normal population, such as community surveys [NOR]; (b) studies based on samples drawn from population with needs, such as patients with chronic illness [NEEDS]; (c) studies based on samples drawn from the helping professionals, such as nurses and clinicians [PROF]; and (d) studies based on samples drawn from the caregivers taking care of people with special needs [CARE]. In the sixth dimension, issues addressed in the empirical studies were classified into two categories: (a) studies about the development of assessment tools [AT] and (b) studies about the application of measurement tools [MT]. Finally, a dimension illustrating other countries (i.e., studies that included other countries for comparison or participants of other countries were included in the sample population) was included.

There are several points that should be noted regarding the present classification scheme. First, as the reviewed papers could appear in more than one database, the final count of papers reviewed (i.e., N = 373) is not equivalent to the sum of the total count of each database. Second, for the empirical studies, only classifications under methodology and issue addressed are mutually exclusive (i.e., QT + QL + MIX = E; AT + MT = E), while more than one classification in a single study under scope, stages of life span, and sample population is possible.

## RESULTS

According to the classification scheme used to categorize the studies about the QOL in Hong Kong, several phenomena could be observed from the findings (see Table 1 and Table 2). First, in the type dimension, the majority of studies about the QOL in Hong Kong were empirical in nature; 339 out of 373 (i.e., 90.88%) papers consisted empirical studies. Second, two major observations in the scope dimension could be highlighted: (a) the number of empirical studies using micro indicators (N = 332) was much greater than the number of empirical studies utilizing macro indicators (N = 7), and (b) among the studies addressing the issue of well-being, personal well-being (N = 325) was most commonly addressed in the empirical studies, followed by family well-being (N = 215), and the least addressed was societal well-being (N = 101). Third, quantitative approach was the most commonly adopted methodology among the empirical studies (N = 294), while the number of empirical studies utilizing qualitative approach and the mixed-method approach were 29 and 16, respectively. Fourth, as for the stages of life span concerned in the empirical studies, the number of studies addressing adults (i.e., adulthood and aged stages, N = 222 and N = 169, respectively) was much greater than the number of studies addressing the adolescence stage (N = 44), childhood stage (N = 16), and the infant stage (N = 3). Fifth, among the four categories in the dimension of sample population, empirical studies based on samples drawn from population with needs had the highest number (N = 246), while the number of empirical studies based on the samples drawn from the normal population followed (N = 89). The number of empirical studies based on samples drawn from the helping professionals and caregivers were the least, with five and six studies, respectively. Sixth, as for the issues addressed in the empirical studies, a larger number of studies were related to the

**TABLE 1**
**Classification of Studies about the QOL in Hong Kong in Three Dimensions (Type, Scope, and Methodology)**

| Database | Total | Type | | Scope | | | | Methodology | | |
|---|---|---|---|---|---|---|---|---|---|---|
| | | T | E | Micro Indicators | | | Macro Indicators | QT | QL | MIX |
| | | | | Per WB | Fam WB | Soc WB | | | | |
| ***ALL*** | ***373*** | ***34*** | ***339*** | ***325*** | ***215*** | ***101*** | ***7*** | ***294*** | ***29*** | ***16*** |
| PsycINFO | 121 | 19 | 102 | 95 | 71 | 45 | 4 | 89 | 6 | 7 |
| Sociological Abstracts | 34 | 4 | 30 | 26 | 20 | 18 | 4 | 26 | 3 | 1 |
| Social Sciences Citation Index | 199 | 12 | 187 | 180 | 125 | 50 | 3 | 166 | 13 | 8 |
| MEDLINE | 174 | 6 | 168 | 164 | 110 | 52 | 1 | 149 | 13 | 6 |
| CINAHL | 95 | 3 | 92 | 91 | 63 | 26 | 1 | 76 | 9 | 7 |
| Others* | 29 | 7 | 22 | 18 | 12 | 11 | 4 | 19 | 2 | 1 |

*Note*: T: theoretical or conceptual studies; E: empirical studies; Per WB: qualitative approach; personal well-being; Fam WB: family well-being; Soc WB: societal well-being; QT: quantitative approach; QL: MIX: mixed-methods approach.

* ERIC, Educational Full Text, and Social Work Abstracts.

**TABLE 2**
**Classification of Studies about the QOL in Hong Kong in Four Dimensions (Stages of Life Span, Sample Population, Issue Addressed, and Comparison with Other Countries)**

| Database | Stages of Life Span | | | | | | Sample Population | | | | Issue Addressed | | Comparison with Other Countries |
|---|---|---|---|---|---|---|---|---|---|---|---|---|---|
| | INFANT | CHILD | ADOL | ADU | AGED | ALL | NOR | NEEDS | PROF | CARE | AT | MT | |
| ***ALL*** | ***3*** | ***16*** | ***44*** | ***222*** | ***169*** | ***15*** | ***89*** | ***246*** | ***5*** | ***6*** | ***65*** | ***274*** | ***25*** |
| PsycINFO | 0 | 4 | 18 | 63 | 47 | 8 | 31 | 70 | 1 | 0 | 29 | 73 | 8 |
| Sociological Abstracts | 0 | 0 | 7 | 9 | 15 | 4 | 22 | 7 | 0 | 1 | 2 | 28 | 3 |
| Social Sciences Citation Index | 1 | 4 | 19 | 131 | 98 | 9 | 51 | 138 | 3 | 2 | 32 | 155 | 10 |
| MEDLINE | 3 | 13 | 22 | 118 | 90 | 6 | 33 | 131 | 2 | 3 | 40 | 128 | 12 |
| CINAHL | 0 | 0 | 3 | 59 | 61 | 1 | 12 | 77 | 0 | 3 | 12 | 80 | 1 |
| Others* | 0 | 0 | 4 | 6 | 9 | 4 | 15 | 6 | 1 | 0 | 5 | 17 | 3 |

*Note*: INFANT: infant stage (0–2); CHILD: childhood stage (3–11); ADOL: adolescence stage (12–17); ADU: adulthood stage (18–59); AGED: aged stage (60+); ALL: all stages; NOR: normal population; NEEDS: population with needs; PROF: helping professionals; CARE: caregivers; AT: development of assessment tools; MT: application of measurement tools.

* ERIC, Educational Full Text, and Social Work Abstracts.

application of the measurement tools (N = 274) as compared to the studies addressing the development of assessment tools (N = 65). Last, but not least, there were relatively few studies that included information of other countries for comparison or with foreign participants included into the sample population.

In addition to the above findings of the overall results, several other observations could be highlighted from the results of the various databases (see Table 3 and Table 4). The databases could be divided into two broad categories: (a) social sciences databases (i.e., PsycINFO, Sociological Abstracts, Social Sciences Citation Index, ERIC, Educational Full Text, and Social Work Abstracts), and (b) medicine and health sciences databases (i.e., MEDLINE and CINAHL). In general, empirical studies were more commonly carried out than theoretical or conceptual studies in both broad kinds of databases, however, the number of the latter type of studies were slightly higher in social sciences databases (i.e., 9.90%) as compared with that of the medicine and health sciences databases (i.e., 3.70%). As for the scope dimension, despite the minimal number of empirical studies addressing macro indicators, only one empirical study was found in the medicine and health sciences databases. Furthermore, the medicine and health sciences databases concentrated more on empirical studies based on samples drawn from the population with special needs as compared to social sciences databases (i.e., 79.81 vs. 69.32%).

**TABLE 3**
**Classification of Studies about the QOL in Hong Kong in Three Dimensions (Type, Scope, and Methodology) According to Two Broad Categories of Databases (Social Sciences Databases and Medicine and Health Sciences Databases)**

| Database | Total | Type | | Scope | | | | Methodology | | |
|---|---|---|---|---|---|---|---|---|---|---|
| | | T | E | Micro Indicators | | | Macro Indicators | QT | QL | MIX |
| | | | | Per WB | Fam WB | Soc WB | | | | |
| ***ALL*** | ***373*** | ***34*** | ***339*** | ***325*** | ***315*** | ***101*** | ***7*** | ***294*** | ***29*** | ***16*** |
| Social Sciences Databases | 293 | 29 | 264 | 253 | 172 | 82 | 6 | 229 | 22 | 13 |
| Medicine and Health Sciences Databases | 216 | 8 | 208 | 204 | 134 | 60 | 1 | 181 | 17 | 10 |

*Note*: Social Sciences Databases: PsycINFO, Sociological Abstracts, Social Sciences Citation Index, ERIC, Educational Full Text, and Social Work Abstracts; Medicine and Health Sciences Databases: MEDLINE and CINAHL.

**TABLE 4**
**Classification of Studies about the QOL in Hong Kong in Four Dimensions (Stages of Life Span, Sample Population, Issue Addressed, and Comparison with Other Countries) According to Two Broad Categories of Databases (Social Sciences Databases and Medicine and Health Sciences Databases)**

| Database | Stages of Life Span | | | | | | Sample Population | | | | Issue Addressed | | Comparison with Other Countries |
|---|---|---|---|---|---|---|---|---|---|---|---|---|---|
| | INFANT | CHILD | ADOL | ADU | AGED | ALL | NOR | NEEDS | PROF | CARE | AT | MT | |
| ***ALL*** | ***3*** | ***16*** | ***44*** | ***222*** | ***169*** | ***15*** | ***89*** | ***246*** | ***5*** | ***6*** | ***65*** | ***274*** | ***25*** |
| Social Sciences Databases | 1 | 6 | 32 | 171 | 135 | 14 | 80 | 183 | 4 | 4 | 50 | 214 | 16 |
| Medicine and Health Sciences Databases | 3 | 13 | 23 | 142 | 116 | 6 | 37 | 166 | 2 | 4 | 43 | 165 | 13 |

*Note*: Social Sciences Databases: PsycINFO, Sociological Abstracts, Social Sciences Citation Index, ERIC, Educational Full Text, and Social Work Abstracts; Medicine and Health Sciences Databases: MEDLINE and CINAHL.

## DISCUSSION

The present review shows that the published papers on QOL in Hong Kong are predominantly empirical studies. There are two factors that contribute to this observation. First, most of the databases would readily include empirical papers (i.e., studies with collection of data) than conceptual papers that simply discuss issues related to QOL. Second, as the focus of research training in Hong Kong is more geared toward research methodology (i.e., application of different research methods) rather than conceptual integration, it is not surprising to note this result. As the concept of QOL has a cultural dimension and the Western concept of "satisfaction" may not be totally applicable to Chinese people[1,2,6], further research in the area of conceptual integration of the QOL concept in Chinese people should be done.

Concerning the scope of the published QOL studies, there are several observations that deserve our attention. First, among the micro QOL studies, very few of them have been conducted with reference to the work settings. As Chinese people (particularly Chinese men) attach a high value to occupational identity[7], research on occupational QOL is important. As Hong Kong is a very achievement-oriented society and work-related stress is high[8], further research should be done. In particular, with the intensification of the globalization movement, more research should be carried out to examine how globalization would affect the QOL of the work force in Hong Kong.

Second, with reference to the observation that more studies utilize micro QOL indicators (e.g., psychological well-being and life satisfaction) than macro QOL indicators (e.g., suicide rates) in examining QOL, the relative strengths and weaknesses of these indicators should be realized[1,2]. It is noteworthy that while micro QOL indicators are more sensitive to individual functioning and conditions, it is difficult to compare people across time and place. On the other hand, macro QOL indicators represent more objective measures of QOL, but cannot capture the subjective experience of people.

Third, the findings showed that there were comparatively fewer studies on family well-being and societal well-being as compared to studies on personal well-being. This observation is consistent with the observation of Shek[9] and Siu and Shek[10] that there are few studies on family well-being in Hong Kong. The present findings suggest that researchers in Hong Kong are more interested in conducting research at the personal level than at the family and societal levels. Shek[9] pointed out that there are several justifications for conducting more research on family well-being: (a) Chinese people constitute roughly one-fifth of the world's population, (b) Chinese data are necessary to establish the generalizability of Western theories and concepts, and (c) Chinese family research forms the basis for family service and policy formulation.

Fourth, as far as the methodology of the research intrinsic to the reviewed studies, the findings showed that quantitative strategy is the predominate mode. This observation is not surprising because quantitative research is still the dominant mode[11]. While there are many strengths of quantitative research, its limitations (e.g., inability to capture the subjective experiences of the participants, insensitivity to contextual variations) should be acknowledged. On the other hand, although qualitative research may be able to examine the complexity of the QOL phenomena, the issues of biases should be addressed. Shek et al.[12] suggested several criteria that could be used to evaluate the quality of qualitative research that can serve as useful pointers for QOL researchers.

Fifth, the findings showed that there were comparatively more QOL research studies on adults and aged people as compared to children and adolescents. This finding is consistent with the observation of Shek et al.[1,2] that there are relatively fewer adolescent QOL research studies. There are two possible factors that contribute to this phenomenon. First, there is more focus on the QOL in adults than in children and adolescents. For example, classification of mental disorders is primarily guided by observation of mental dysfunction in adults. Second, the relatively stronger emphasis on parent-centeredness than child-centeredness in the Chinese culture may also contribute to this observation.

Sixth, as far as sample populations are concerned, the present findings showed that more studies were based on participants with special needs (e.g., mentally retarded people and patients with chronic illnesses), which were followed by people in the normal population. This observation is reasonable because the QOL concept has been historically related to the life conditions of people with special needs.

On the other hand, the QOL of health professionals and caregivers was comparatively neglected. As burnout among professionals (e.g., teacher burnout) and caregivers is not uncommon[13], more research in this area is needed.

Seventh, there is a lack of comparative data intrinsic to QOL research in Hong Kong, an observation that is consistent with the finding of Shek et al.[1,2]. This is probably due to the fact that there is a lack of assessment tools (e.g., life satisfaction measures) that can be suitably compared across different places. In the long run, there is a need to devise tools and indicators that can be used to compare QOL across different communities. Besides showing the differences between Hong Kong and other societies in QOL measures, comparative findings can also show the unique features of QOL in the Hong Kong society.

There are several limitations of this review. First, only two search terms (“quality of life” and “Hong Kong”) were used in this review. If resource permits, more search terms could be used to conduct the review. Second, it is noteworthy that the use of “quality of life” as a search term in this study may exclude those studies that have studied QOL, but have not used this term in the study. For example, Shek[14] investigated changes in positive youth development without using the term “quality of life”. Shek et al.[15] also examined adolescent suicide in Hong Kong without using the term “quality of life”. Third, additional dimensions (e.g., whether the sample was randomly drawn and whether the measures used were valid and reliable) could be used in future studies to give further information on the methodological attributes of the studies. Finally, the present review is limited to QOL studies in Hong Kong. It would be illuminating if studies in other Chinese communities could be added. Despite these limitations, this review provides a picture on the QOL studies in Hong Kong and the future research directions.

## ACKNOWLEDGMENTS

The preparation for this paper was financially supported by the Wofoo Foundation Limited.
